# First report of primary testicular leiomyosarcoma in two dogs

**DOI:** 10.1186/s12917-023-03658-5

**Published:** 2023-07-31

**Authors:** Rafał Ciaputa, Eleonora Brambilla, Francesco Godizzi, Stanisław Dzimira, Kacper Żebrowski, Małgorzata Kandefer-Gola, Marcin Nowak, Valeria Grieco

**Affiliations:** 1 Department of Pathology, Division of Pathomorphology and Veterinary Forensics, Faculty of Veterinary Medicine, Wroclaw University of Environmental and Life Sciences, C.K. Norwida 31, Wroclaw, 50-375 Poland; 2 Department of Veterinary Medicine and Animal Science, University of Milan, via dell’Università 6, Lodi, 26900 Italy

**Keywords:** Testis, Tumour, Dog, Sarcoma, Immunohistochemistry

## Abstract

**Background:**

Testicular tumours are common in dogs and, among them, interstitial cell tumours, seminomas and sustentacular cell tumours are the most reported. Mesenchymal testicular tumours are rarely reported in humans as in veterinary medicine where only three cases of sarcomas (leiomyomas and leomyosarcomas) have been described in two stallions and in a ram.

**Case presentation:**

The present cases regarded a 12-year-old mixed-breed dog and a 10-year-old American Staffordshire Terrier that underwent bilateral orchiectomy. Formalin fixed testes were referred for histopathological diagnosis. At gross examination, in one of the testes of both dogs, a white, firm and variably cystic testicular mass, effacing and replacing the testicular parenchyma was detected. Samples were collected from both neoplastic and contralateral testes, routinely processed for histology and serial sections were also examined immunohistochemically with primary antibodies against cytokeratins, vimentin, Von Willebrand factor, inhibin-α, α-smooth muscle actin, smooth muscle myosin and desmin. Histopathological features as well as the immunohistochemical results, positive for vimentin, actin, myosin and desmin, confirmed the mesenchymal origin and the myoid phenotype of both testicular tumours supporting the diagnoses of leiomyosarcoma.

**Conclusions:**

To the authors knowledge these are the first cases of primary testicular sarcoma reported in the canine species. However, even rare, these tumours deserve to be considered in routine diagnosis when a testicular spindle cell tumour is observed. The immunohistochemical panel applied was useful to distinguish the present tumours from undifferentiated Sertoli cell tumours confirming the diagnosis of leiomyosarcoma.

## Background

Testicular tumours are common in dogs while, except for the horse, they are very rare in other domestic species [1]. The classification of testicular tumours in animals presented by the World Health Organization listed as the most common testicular tumours: interstitial cell tumours (Leydig cell tumours), seminomas and sustentacular cell tumours (Sertoli cell tumours) [2]. Other neoplastic lesions are recorded as less frequent such as: mixed germ cell-sex cord-stromal tumour, teratoma and embryonal carcinoma [2].

Testicular tumours develop from cells that can further differentiate along the gonadal lineage (seminomas) or develop into populations of pluripotent cells (non-seminomas). Pluripotent cells may remain at the stage of undifferentiated cells (embryonal carcinoma), differentiate into extra-embryonic tissues (endodermal sinus tumour, yolk sac tumour, malignant choriocarcinoma) or somatic (teratoma) [3].

Sarcomas are a heterogeneous group of malignant tumours of mesenchymal origin. About 80 per cent of sarcomas originate from soft tissue and the rest arise from the bone. The histopathological spectrum of sarcomas is broad because the embryonic mesenchymal cells from which they arise can mature into, among others, striated and smooth muscles, adipose and fibrous tissue, bone and cartilage [4, 5]. For a correct diagnosis discriminating among these tumours and to differentiate them from epithelial derived ones, immunohistochemical panels including various antibodies are generally used in routinary histopathologic diagnosis [5]. In these panels, cytokeratins and vimentin are applied to discriminate from epithelial and mesenchymal origin of the tumours. Moreover, antibodies directed against α-SMA actin, myosin, and desmin are commonly included to discriminate among mesenchymal tumours deriving from smooth and skeletal muscles while antibodies for Von Willebrand factor are used to recognize haemangioma/haemangiosarcomas [5].

In humans, testicular sarcomas are also very rarely noticed as primary lesions. The majority of publications regarding these tumours are case reports, and leiomyosarcoma seems to be the most commonly diagnosed sarcoma subtype [6–9]. Long-term anabolic steroid assumption, chronic inflammation of the testis, testicular germ cell tumours and testicular field radiation for treatment of leukaemia have been proposed as predisposing factors for testicular leiomyosarcoma development in young human patients [7–10]. To the authors’ knowledge, there is only one large cohort study reporting clinical characteristics and prognostic factors in 158 cases of testicular sarcomas in humans [4]. In this study, localized and distant metastases occurred in 20.13% and 18.18% of patients with testicular sarcoma, while 61.69% had no metastasis. Unfortunately, in the study, tumour histologic subtypes and their distribution among the caseload were not reported [4].

As far as animal testicular mesenchymal tumours are concerned, to the authors’ knowledge, there are few reported cases in veterinary medicine: in two stallions and in a ram [11–13]. Interestingly, all the cases were of myoid phenotype (leiomyomas and leiomyosarcomas)[11–13]. Because mesenchymal testicular tumours are so rare in human as in animal species, the aim of the present report was to histologically describe and immunohistochemically characterize two cases of primary testicular sarcoma in dogs.

## Case presentations

Two dogs, a 12-year-old mixed-breed dog (case one) and a 10-year-old American Staffordshire Terrier underwent bilateral orchiectomy. The two formalin-fixed pairs of testes were received for histology. For each pair of testes, it was impossible to determine which was the left and which the right gonad, however, in each pair of testes one was apparently normal while the other testis, on the cut section, was completely effaced by a firm, greyish-white tumour. In case one, the tumour was 15 cm x 9 cm and irregularly shaped while, in case two, the tumour was multilobulated, 18 × 12 cm in size. Within both tumours, a cystic space, filled with cloudy fluid, was recognizable and in case 2 a single focal haemorrhage was also present. In both cases, the dogs had no previous cancer history, and clinical examination and diagnostic imaging did not identify the presence of primary tumours or metastases in other body districts at the time of orchiectomy. For both the dogs the surgery, without any adjuvant therapy, was curative but, after three years, the mixed breed dog (case one) had no distant metastases and died of a cardiac disorder while the American Staffordshire Terrier (case two) is currently alive, without local relapse and/or distant metastases at the time of manuscript submission (2,5 years after orchiectomy).

For both cases described, samples from each testis (neoplastic and normal) were routinely processed for histology. From paraffin blocks serial 4-µm thick sections were obtained. One section was stained with haematoxylin and eosin and the other sections were examined immunohistochemically.

Immunohistochemistry detection was performed using avidin-biotin-peroxidase complex method (VECTASTAIN® ABC Kit, Vector Laboratories) with a panel of primary antibodies reported in Table 1. The immunolabeling reaction was developed using 3,3’-diaminobenzidine chromogen (DAB Peroxidase Substrate Kit, Vector Laboratories). As negative controls, to evaluate the specificity of the markers, according to the standards for validation of immunohistochemical assays by Hewitt et al., 2014, replicate sections were incubated with isotype-specific immunoglobulins [14]. As these immunoglobulins were from the same species in which the primary antibody was produced, mouse immunoglobulins were used for replacing the primary monoclonal antibodies (Table 2) while rabbit immunoglobulins replaced the polyclonal antibody directed against Von Willebrand Factor. Immunohistochemistry was also performed on serial sections of the normal testis, of each pair of testes, which contained positive controls of the immunoreaction: rete testis for cytokeratins, interstitial fibroblasts for vimentin, endothelial cells for Von Willebrand factor, vessel walls for alpha smooth muscle actin (α-SMA), smooth muscle myosin (SMM) and desmin. Interstitial Leydig cells were used as positive control for inhibin α [14, 15].

**Table 1:** Antibodies Used in IHC (Antigen Retrieval, Dilutions, and Sources)

ANTIBODY	CLONE	SUPPLIER	ANTIGEN RETRIVAL	DILUTION
Cytokeratin	MNF 116	Dako, Carpinteria, CA, USA	30 min. in pH 6.0, citrate buffer, Leica Bound-Max	1:75
Vimentin	3B4	Dako, Carpinteria, CA, USA	30 min. in pH 6.0, citrate buffer, Leica Bound-Max	1:100
Desmin	D33	ScyTek Laboratories, Inc., USA	20 min. in pH 9.0, EDTA buffer, Leica Bound-Max	Ready to use
α-Smooth Muscle Actin	1A4	Sigma-Aldrich, USA	20 min. in pH 6.0, citrate buffer, Leica Bound-Max	1:1000
Smooth Muscle Myosin	SMMS-1	Sigma-Aldrich, St. Louis, MO.	20 min. in pH 6.0 citrate buffer, Leica Bound-Max	1:400
Inhibin α	R1	Serotec Corporation Oxford, UK	MW 650 W, 10 min. in pH 6.0 citrate buffer	1:40
Von Willebrand Factor	Polyclonal	Dako, Carpinteria, CA, USA	20 min. in pH 9.0, EDTA buffer, Leica Bound-Max	1:800

Immunohistochemical results were assessed using a modified semiquantitative IRS scale according to Remmele et al. [16], reported in Table 2.

**Table 2:** Remmele et al., 1987 [16], semiquantitative scale applied for the immunohistochemical evaluation of the two cases examined

Percentage of positive cells	Points	Immunohistochemical reaction	Points
0	0	Absent	0
1–10%	1	Weak	1
11–50%	2	Moderate	2
51–80%	3	Intense	3
>80%	4		

**Fig. 1:**
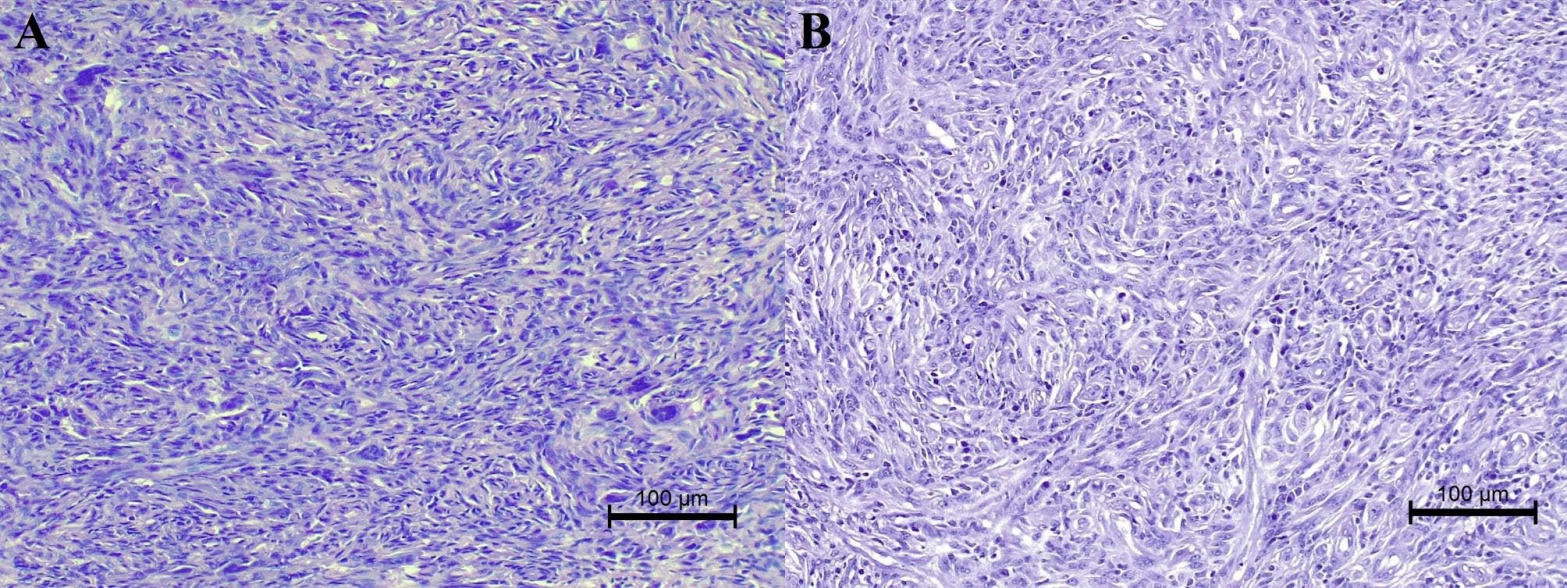
**A** Testicular tumour of case one, 40x, Hematoxylin and Eosin stain. Spindle cells arranged in short interlacing-interwoven bundles and occasionally in a storiform or storiform fingerprint pattern. **B** Testicular tumour of case two, 40x, Hematoxylin and Eosin stain. Neoplastic cells were spindle, with indistinct cell borders and intermediate to high nuclear to cytoplasmic ratio

Tumours were composed of spindle cells arranged in short interlacing/interwoven bundles in case 2 (Fig. 1B) and, in case 1, occasionally in a storiform or fingerprint pattern (Fig. 1A). Neoplastic cells, ranging from 25 to 30 μm in length, had indistinct cell borders, intermediate to high nuclear to cytoplasmic ratio, moderate amount of eosinophilic fibrillary cytoplasm and round to oval vesicular nuclei, 10–20 μm in diameter, with finely granular chromatin and one prominent nucleolus. Anisokaryosis and anisocytosis were moderate in both cases while mitoses were seventeen in 2.37mm^2^ in the first case and four in the second one. Scattered multinucleated neoplastic giant cells were also present in case one. Both tumours were infiltrated by multifocal aggregates of small mature lymphocytes and plasma cells, which in the second case were variably arranged in follicular structures. Neoplastic tissue, in case two in particular, was dissected by multifocal haemorrhages surrounded and infiltrated by numerous haemosiderophages. Within the second tumour a large area of colliquative necrosis, associated with cholesterol clefts and multifocal haemorrhages, was also present.

Tissue samples showed the expected markers expression in positive controls, demonstrating that samples were correctly reacting. Immunohistochemical results are summarized in Table 3. Both tumour were negative for both cytokeratins and von Willebrand factor, excluding an epithelial and endothelial origin respectively. The negative immunoreaction for inhibin-α excluded an origin of the tumour from interstitial Leydig cells. Moreover, since it has been demonstrated that neoplastic canine Sertoli cells are positive for inhibin α, the negative reaction in the present cases allowed to exclude the origin of both tumours from these cells [17].

**Fig. 2:**
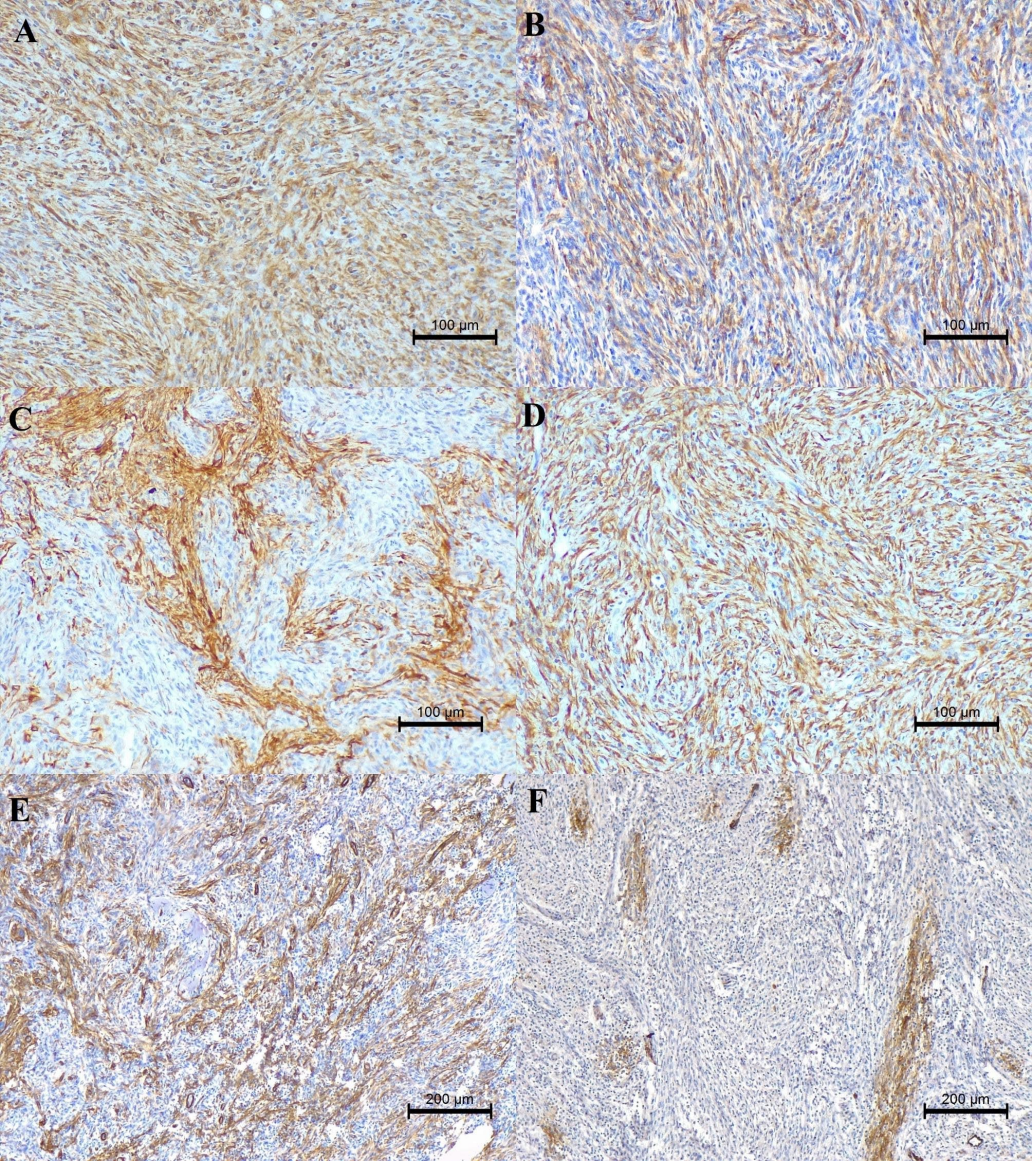
Immunohistochemical characteristics of testicular tumours. **A**. Intense cytoplasmic vimentin reaction, 40x. **B** Moderate cytoplasmic α-SMA reaction, 40x. **C**; **D** Moderate cytoplasmic response for Desmin, 40x. **E**; **F** Moderate cytoplasmic response to SMM, 40x

Vimentin was diffusely and highly expressed in 100% of neoplastic cells in both cases (Fig. 2A) supporting the mesenchymal origin of both tumour. Regarding other specific markers, tumours reacted to α-SMA (Fig. 2B), desmin (Fig. 2C and D) and SMM (Fig. 2E F), suggesting a smooth muscle origin for both neoplasms.

Currently, given their exceptional rarity, there is no specific grading system for canine testicular sarcomas. We therefore applied the one commonly used to grade canine soft tissue sarcomas which considers tumour differentiation, mitotic count and percentage of necrosis [18, 19]. According to this system, case 1 was considered undifferentiated (differentiation score 3), with seventeen mitoses in 2.37mm^2^ (mitotic score 2) and with no areas of necrosis (necrosis score 0) receiving a total score of 5 and thus assigned grade 2. Case 2 was undifferentiated too (differentiation score 3), with four mitoses in 2.37mm^2^ (mitotic score 1) and a large, albeit ≤ 50% of all the analysed sections, area of necrosis (necrosis score 1), and based on the total score [5] was assigned grade 2.

**Table 3:** Immunohistochemical results, considering the percentage of positive cells (points of percentage of positive cells) and the intensity of the immunohistochemical reaction, according to the scale from Remmele et al., 1987 [16]

Antibody	Case 1		Case 2	
	Points **of percentage of positive cells**	**Points of Immunohistochemical reaction intensity**	**Points of percentage of positive cells**	**Point of Immunohistochemical reaction intensity**
Cytokeratin	0	0	0	0
Vimentin	4	3	4	3
Desmin	3	2	4	3
Myosin	3	3	1	3
α-SMA	4	3	4	2
Inhibin	0	0	0	0
Von Willebrand factor	0	0	0	0

## Discussion and conclusions

The immunohistochemical results confirmed the mesenchymal origin of both testicular tumours examined and supported the diagnoses of leiomyosarcoma [14]. Moreover, the positive immunolabelling for markers typical of the muscle differentiation and the co-expression of desmin, α SMA and SMM were consistent in both cases with the diagnosis of leiomyosarcoma [20]. However, considering the expression of smooth muscle markers, but also the presence of scattered neoplastic multinucleated giant cells, the diagnosis of undifferentiated pleomorphic sarcoma could not be excluded for case 1 [20]. Interestingly, although neoplastic multinucleated giant cells were negative for muscular markers, this feature has been already reported in human leiomyosarcomas, supporting this diagnosis [21]. Additionally, the differential diagnosis of myofibroblastic sarcoma should have been considered solely based on the morphological features of both testicular tumors [22]. However, both cases described here showed strong and diffuse desmin staining, a finding which should be always considered indicative of true smooth muscle differentiation [23]. Indeed, although rare canine myofibroblastic fibrosarcomas expressing desmin are reported, this muscle cytoskeletal protein is considered virtually absent in myofibroblasts, thus reasonably excluding a diagnosis of myofibroblastic sarcoma in the cases described here [22, 23].

To the authors’ knowledge these are the first cases of primary testicular leiomyosarcoma reported in the canine species and the collected literature showed that such condition, even rare, seems more often described in humans than in animals. However, interestingly, all the few animal cases described in literature seem to derive from smooth muscle cells [11–13]. It is also worth noting that, among these tumours, only one detected in a stallion was diagnosed as leiomyosarcoma, while the other equine case and the occasional one described in ruminants were diagnosed as leiomyomas [11–13]. In our cases, the severe effacement of the testicular parenchyma, which in both cases was totally substituted by the neoplastic mass together with the presence of anisokaryosis and anisocytosis, vesicular nuclei, numerous mitoses in case 1 and large necrotic areas in case 2, led us to indicate both tumours as malignant.

However, independently from their biological behaviour (benign or malignant), mesenchymal tumours of the testis are exceedingly rare in animals, as they are not reported in dogs or mentioned in past or recent epidemiological studies based on a huge number of canine testicular tumours [2, 24–26]. Moreover, even if studies demonstrated that the number of testicular tumours is constantly increasing in canine species, testicular sarcomas were not observed, proving their rare occurrence ( 2, 24–26).

Human testicular leiomyosarcomas are subdivided into paratesticular and intratesticular with paratesticular tumour being more common [6]. Paratesticular sarcomas can arise mainly from the epididymis, spermatic cord, and tunica vaginalis [27]. In a rare case of paratesticular leiomyosarcoma recently described in a young dog, the epididymis and testicular surrounding tissues were affected while the testis was still clearly recognizable [28]. In our cases, conversely, the complete replacement of the testicular parenchyma and the fact that both tumours were limited to the testis confirmed the intratesticular origin of the tumour. A diagnosis of angioleiomyosarcoma could not be ruled out, even though the supposed origin from vessel walls was not evident in either tumour. Both tumours were grade II and after surgery neither case presented recurrence and/or local or distant metastasis. However, studies on a larger number of cases are needed to better characterise testicular soft tissue sarcomas in dogs and to understand their metastatic potential.

Immunohistochemistry is commonly used in oncopathology, however, the accuracy of the diagnosis may depend on the number of the markers employed, so that the use of a large panel of antibodies, as in the present study, is recommended to delineate the cell origin of the tumour.

## Data Availability

The datasets used and/or analysed during the current study are available from the corresponding author on reasonable request.
